# Detection of Microaneurysms in Fundus Images Based on an Attention Mechanism

**DOI:** 10.3390/genes10100817

**Published:** 2019-10-17

**Authors:** Lizong Zhang, Shuxin Feng, Guiduo Duan, Ying Li, Guisong Liu

**Affiliations:** 1 School of Computer Science and Engineering, University of Electronic Science and Technology of China, Chengdu 611731, China; 2 School of Computer Science, Zhongshan Institute, University of Electronic Science and Technology of China, Zhongshan 528400, China

**Keywords:** MA detection, fundus image, attention mechanism

## Abstract

Microaneurysms (MAs) are the earliest detectable diabetic retinopathy (DR) lesions. Thus, the ability to automatically detect MAs is critical for the early diagnosis of DR. However, achieving the accurate and reliable detection of MAs remains a significant challenge due to the size and complexity of retinal fundus images. Therefore, this paper presents a novel MA detection method based on a deep neural network with a multilayer attention mechanism for retinal fundus images. First, a series of equalization operations are performed to improve the quality of the fundus images. Then, based on the attention mechanism, multiple feature layers with obvious target features are fused to achieve preliminary MA detection. Finally, the spatial relationships between MAs and blood vessels are utilized to perform a secondary screening of the preliminary test results to obtain the final MA detection results. We evaluated the method on the IDRiD_VOC dataset, which was collected from the open IDRiD dataset. The results show that our method effectively improves the average accuracy and sensitivity of MA detection.

## 1. Introduction

Automated medical data processing is a current trend in bioinformatics analysis that is beneficial for disease detection and diagnosis. Diabetic retinopathy (DR) is one of the most serious complications of diabetes mellitus and is a major cause of blindness worldwide [1,2]. No apparent clinical symptoms exist during the early stage of DR, but the disease causes fine lesions to form on the retina and eventually leads to blindness. Therefore, early diagnosis and treatment of DR are highly important. However, the existing DR screening method relies on professional ophthalmologists to assess colour retinal fundus images, a procedure that requires a high level of professional expertise and has low screening efficiency. As the incidence of diabetes increases, the necessary medical resources are becoming increasingly scarce.

Thus, similar to other automated disease detection applications [3,4], small object detection in fundus images is vital for DR screening. In the early stages of DR, subtle changes occur in the retina, causing capillaries to expand and form microaneurysms (MAs) [5]. On a fundus image, these MAs usually appear as dark red dots with diameters ranging from 15 μm to 60 μm. These dots constitute less than 0.15% of the total pixels. Therefore, they are aptly termed “small objects”. To date, many studies have achieved progress in the machine-based detection of general objects, but small object detection is still a difficult task in the field of image processing.

Many scholars have conducted research on common object detection, including small target recognition. However, few researchers have used deep learning to detect MAs in fundus images. The current object detection algorithms cannot effectively use the microtarget features extracted in the feature layer; thus, they are unsuitable for use in detecting MAs.

Motivated by the above issues, this paper proposes a novel small object detection method based on a multilayer attention mechanism and spatial confidence. First, the multilayer attention mechanism effectively utilizes useful information for small object detection in feature maps extracted from preprocessed images, causing more attention to be paid to areas in which such objects may appear in the feature maps. The output of this process forms the preliminary detection results. Then, the reliability of the preliminary test results is judged based on spatial confidence discrimination, and false positive objects are screened out to improve the object detection accuracy.

The contributions of this paper are as follows:

1. This paper proposes a fundus image quality equalization method for preprocessing and slicing fundus images; then, based on the proposed method, we construct the fundus dataset IDRiD_VOC.
2. This paper proposes a feature fusion algorithm based on a multilayer attention mechanism that includes both feature layer and channel fusion. Using this algorithm, the feature layer with obvious small target features can be selected more accurately, and the preliminary detection of small target can be realized.
3. This paper proposes a confidence discrimination method based on spatial positional relationships, including target and blood vessel distance calculations, target confidence scores, and a voting mechanism. We use this method to perform a secondary screening of the small target results of the preliminary detection process. Our confidence discrimination method improves the detection sensitivity while ensuring sufficient detection accuracy.

The rest of this article is organized as follows. Section 2 first introduces the dataset and works related to our research, then Section 2.3 describes the detection methods proposed in this paper in detail, Section 3 evaluates the effectiveness of the proposed framework, and Section 4 concludes the paper.

## 2. Materials and Methods

### 2.1. Dataset

**Figure 1:**
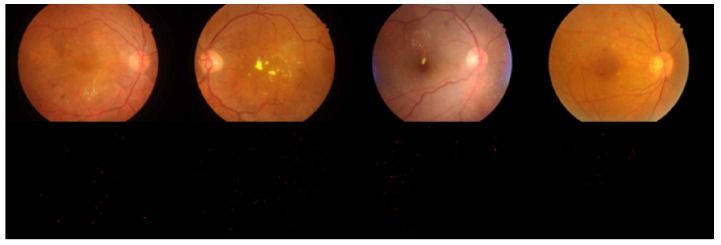
IDRiD fundus images and microaneurysm segmentation maps.

In this study, the microaneurysm part of the IDRiD dataset [6] was used as the original dataset. The IDRiD images were acquired using a Kowa VX-10 alpha digital fundus camera (Nanded, Maharashtra, India) with a 50-degree field of view (FOV), and all are centred near the macula. The fundus images have a resolution of 4288 × 2848, and the images are in JPEG format. The dataset used here consists of 81 images for MA detection and contains a corresponding binary MA label map for each image. Examples are shown in Figure 1, in which the first row of images shows colour retinal fundus images, and the second row shows the MA segmentation maps corresponding to the first row of images.

**Figure 2:**
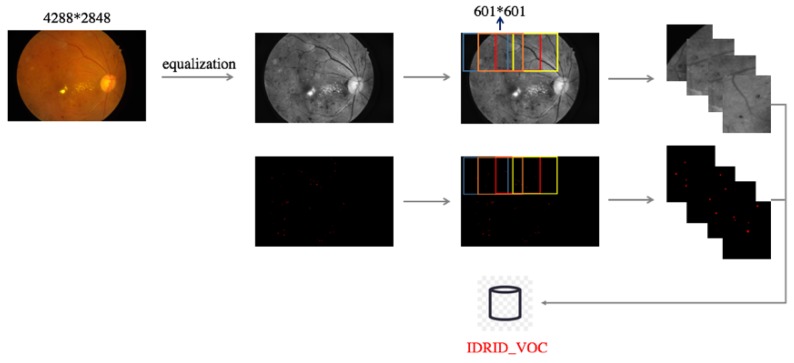
Construction of IDRiD_VOC using the sliding window method. The equalized images and segmentation maps were both sliced. After slicing, some useless image blocks and the corresponding segmentation map blocks were discarded, such as corner patches containing no meaningful information.

**Figure 3:**
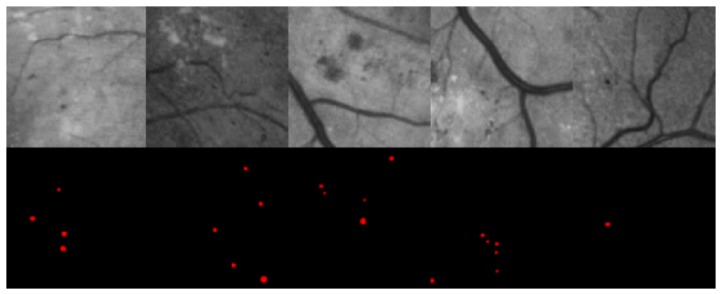
Image blocks and MA segmentation maps. After image preprocessing, each image in IDRiD_VOC contains at least one target MA object, and the dataset includes no large areas of all black blocks.

The image quality equalization method described in Section 2.3.1 was used to process the dataset described above. Then, image blocks were obtained by applying a sliding window to each processed image, as shown in Figure 2 and Figure 3. Figure 2 illustrates the sliding window process applied to the equalized images, and Figure 3 shows the results, where the first row shows the image blocks, and the second row shows the MA segmentation maps corresponding to the image blocks.

Using the Pascal VOC dataset format, a dataset of eye fundus images was produced using the LabelImg tool (https://github.com/tzutalin/labelImg). The final dataset includes 3583 image blocks, corresponding XML annotation files, and related texts (used to record which images belong to the training and test sets). Specifically, the training set includes 3200 patches, and the test set contains 383 patches. Henceforth, this dataset is simply referred to as IDRiD_VOC.

### 2.2. Related Work

#### 2.2.1. Current Object Detection Methods

At present, common object detection methods include both traditional machine learning methods and deep learning methods. Traditional object detection methods are mostly based on a sliding window model, and they extract and match manually designed features. Representative traditional methods include the AdaBoost algorithm with Haar features [7,8,9,10], the support vector machine (SVM) algorithm with histogram of oriented gradients (HOG) features [11,12,13], and the data projection method (DPM) [14,15,16] algorithm. Object detection algorithms are based on deep learning extract features autonomously using a deep neural network. Representative deep learning methods include the various versions of region-based convolutional neural networks (R-CNNs) [17,18,19], the various versions of You Only Look Once (YOLO) [20,21,22], and the Single Shot Detector (SSD) [23]. The traditional methods have various shortcomings: they are overly simplistic, require complex calculations, have insufficient applicability, and suffer from poor detection accuracy and speed. By contrast, deep learning methods have shown greater advantages in these respects.

Small object detection methods based on deep learning mostly use the image pyramid method for feature fusion, which is widely used in digital signal analysis [24,25]. The authors of [26] proposed a general strategy for selecting the template size to be used in detection. First, the input image is transformed into different scales to construct an image pyramid. These images are later used as input to a convolutional neural network (CNN) for training. Then, the feature size that is best suited to the small target detection task is selected to improve the accuracy of small target detection. The authors of [27] proposed a feature fusion method for SSD whose core idea is to fuse multilevel features to acquire contextual information. This method fuses the features from layers conv4_3 and conv5_3 and then uses a high-level feature map to enhance the semantic information of the low-level feature maps. This approach increases the accuracy of small target detection. In [28], a feature pyramid method was proposed. By combining high-level features with low-level features from earlier layers through downsampling, feature layers with different resolutions can be endowed with rich semantic information, and detection can be performed based on each layer separately. On the basis of the SSD and Feature Pyramid Network (FPN) approaches, the authors in [29] added shallow features to the feature fusion queue for small target detection.

MA detection methods based on deep learning mostly employ image segmentation or pixel-by-pixel classification. The model proposed in [30] uses a single CNN to segment pathological features such as exudate, hemorrhage and MA features but does not consider the specificity of different lesions. The authors of [31] were the first to perform vascular removal during the extraction of candidate MAs. However, the incomplete removal of blood vessels caused the model to easily confuse the remaining blood vessel regions with real MAs. The authors of [32] proposed a method for using a CNN’s ability to output probabilities to output a class probability for each pixel. This method was able to simultaneously detect exudation, hemorrhage, and MAs based on the output probability map. In [33], the use of geometrical properties based on connected regions was shown to enable the distinction between lesion pixels and non-lesion pixels. However, pixel-by-pixel classification methods are computationally intensive, and they lack consideration of the surrounding environment.

All of the deep-learning-based object detection methods described above are unsuitable for use in the MA detection task. In [17,18,19,20,21], image features were extracted by a CNN, followed by a regression analysis and a confidence calculation of the prediction frame based on the features obtained from the last layer. In [23,26], multiscale targets were detected by inputting feature maps or images of different scales into the network. However, none of these methods are able to focus on the feature information corresponding to small targets; they ignore the correlations between feature layers at different levels, which can easily lead to a loss of shallow features and a reduction in the ability of the model to detect small targets. Several studies [22,27,28,29] have attempted to fuse different feature layers to obtain more abundant image information. For the merging of different pyramid feature layers, convergence schemes such as concat (combination of channels) and eltsum (merging of feature maps) are usually considered. However, when the features are combined, all the information is processed uniformly, and the relative importance of different feature layers and different receptive field information is not considered. At the same time, the idea of SSD, which uses deeper feature maps (conv9-11) for prediction, has been widely adopted. However, unless lower-layer feature maps are included, this approach cannot improve the detection of small targets.

To make the model pay more attention to image features that are specifically related to small target detection, an attention mechanism is adopted in this paper.

#### 2.2.2. Attention Mechanism

Attention mechanisms were originally developed to solve the problem of model distraction during machine translation. Bahdanau et al. [34] were the first to use an attention mechanism to achieve differences in the contributions of different input sequences to the output sequence. Subsequently, attention mechanisms have undergone continuous development, and they are widely used in various natural language processing tasks [35,36,37,38,39].

Attention mechanisms are also commonly used in image processing, especially in image classification [40,41,42], semantic segmentation [43,44], object detection, and similar tasks. An attention network was proposed in [45] that provides a quantitative weak direction for the object search, ensures that the prediction set will iteratively converge to an accurate object boundary frame, and achieves more accurate object detection. Based on Faster R-CNN, Dai et al. [46] proposed position-sensitive region-of-interest (RoI) pooling, a type of attention mechanism that incorporates spatial information to solve the location sensitivity problem in target detection. Liu et al. [47] applied an attention mechanism for the detection of human heads in images for the estimation of population density.

In recent years, attention mechanisms for medical image processing have begun to emerge. Zhang et al. [48] introduced an attention enhancement module (AAS) to assist an attention module in generating a more efficient attention map. The authors of [49] proposed an attention-based CNN for glaucoma detection. Tang et al. [50] proposed an attention mechanism for 3D medical image segmentation, in which a cascaded detection module followed by a segmentation module was applied to produce a set of object region candidates. Nonetheless, very few algorithms have been proposed in which an attention mechanism is applied to MA detection, and this gap in the literature motivates the proposal of our framework.

### 2.3. Methods

Inspired by the concept of attention mechanisms, a new feature fusion network for MA detection is presented to extract features related to small targets. In addition, we note that the spatial relationship between MAs and blood vessels follows a certain distribution; consequently, a spatial voting algorithm is proposed to screen the preliminary detection results.

**Figure 4:**
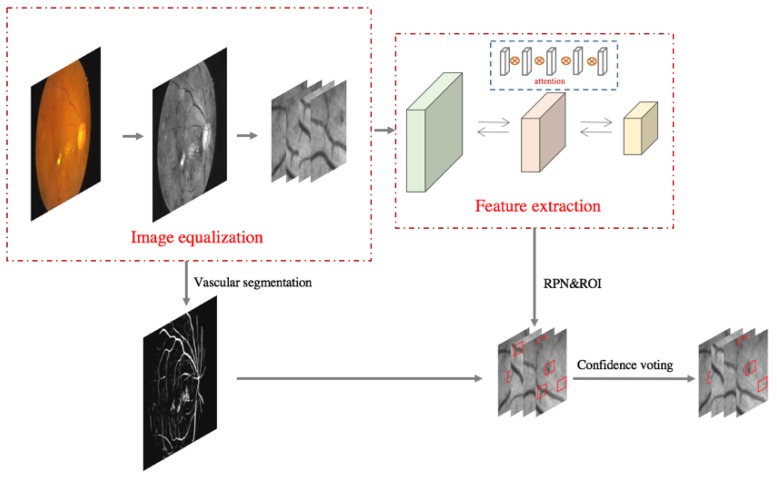
Overview of our algorithm. The details of the image equalization process are shown in Figure 5. The output of feature extraction with the attention mechanism is a fused feature map with 256 channels that is passed to a region proposal network (RPN) along with the region of interest (RoI) for bounding box prediction. Vascular segmentation is conducted on each equalized image to acquire a spatial confidence score for each predicted bounding box; this process is discussed in detail in Section 2.3.7.

The framework of the method proposed in this paper is illustrated in Figure 4. The fundus images are first processed for quality equalization. Then, two operations are applied to each image: one segments the image into a dataset for small object detection, and the other obtains the corresponding vascular segmentation map. The results are input into a basic feature extraction network, and the resulting multilayer feature maps are fused by means of a multilayer attention mechanism. The fused feature map is then input into a region proposal network (RPN) and subsequently classified and regressed to obtain the preliminary small object detection results. Based on the coordinates of the predicted boxes after preliminary detection and the vascular segmentation map, a confidence score is calculated for each prediction frame. Finally, the confidence scores and probability scores are screened twice through majority voting to obtain the final small object detection results.

#### 2.3.1. Fundus Image Quality Equalization

Due to the image acquisition environment and the presence of lesions, fundus images tend to be of inconsistent quality, and small objects may not be obvious in the original images. Therefore, a fundus image quality equalization method is proposed. For example, in Figure 6, the images shown in panels (a) and (b) are too bright and too dark, respectively, due to the imaging environment; consequently, the small object information is blurry and cannot be distinguished. In panel (c), many of the image details are obscured by the large hemorrhage and exudate areas. In our method, blurred images with lesions that are severe enough to cover a large area of the fundus (c) are screened out.

**Figure 5:**
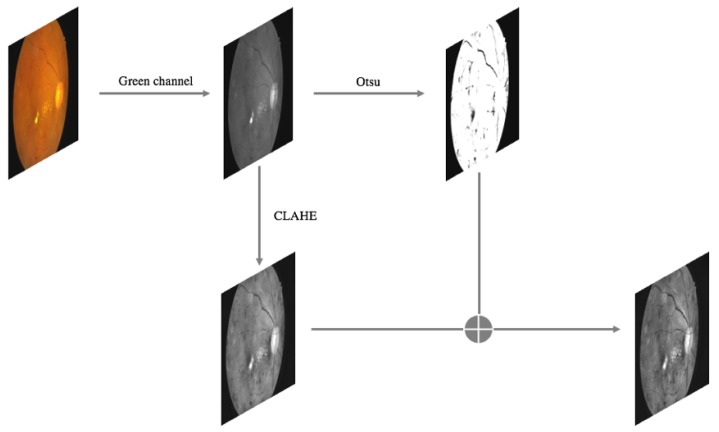
Overview of our image quality equalization method. The green channel of an image shows more texture information than the red and blue channels do [51,52]. The Otsu method is widely used in image processing to emphasize the differences between background and foreground, making it an effective means of enhancing image contrast [53,54,55].

**Figure 6:**
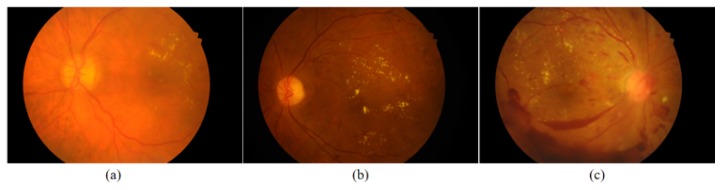
Examples of inconsistent image quality. These images show three types of inconsistency: (**a**) a too-bright image; (**b**) a too-dark image; and (**c**) an image with large hemorrhage and exudate areas. Among these, (**a**,**b**) can be equalized to obtain normal images, while (**c**) will be rejected in our method.

Then, quality equalization is conducted on the remaining images. The specific operational flow is illustrated in Figure 5.

**Figure 7:**
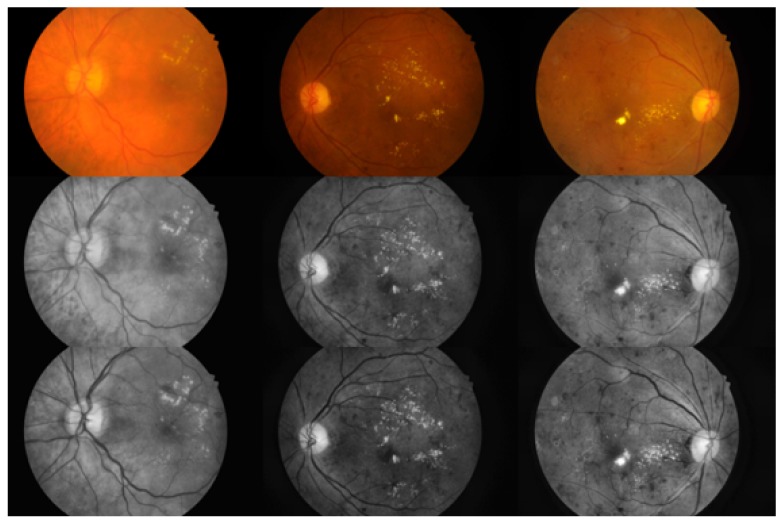
Comparison of our method with typical adaptive histogram equalization. From left to right, the images shown are (**top**) a bright image; (**middle**) a dark image; and (**bottom**) a normal image. It is clear that our method effectively improves the image contrast, which is helpful for MA detection.

First, the green channel of each colour fundus image is extracted in the RGB colour space, which is defined by three chromaticity channels of the red, green and blue additive primaries. Then, the Contrast-Limited Adaptive Histogram Equalization (CLAHE) algorithm is applied to the green channel to enhance the contrast of the fundus image. At the same time, the green-channel image is segmented using the Otsu threshold segmentation algorithm, which divides the image into foreground and background. Finally, the images obtained by using the CLAHE algorithm and the Otsu algorithm are combined to extract the RoI of the fundus image. In Figure 7, the first row shows several original images, the second row shows the results of the adaptive histogram equalization procedure, and the third row shows the results of our algorithm.

#### 2.3.2. Feature Fusion Based on Attention

**Figure 8:**
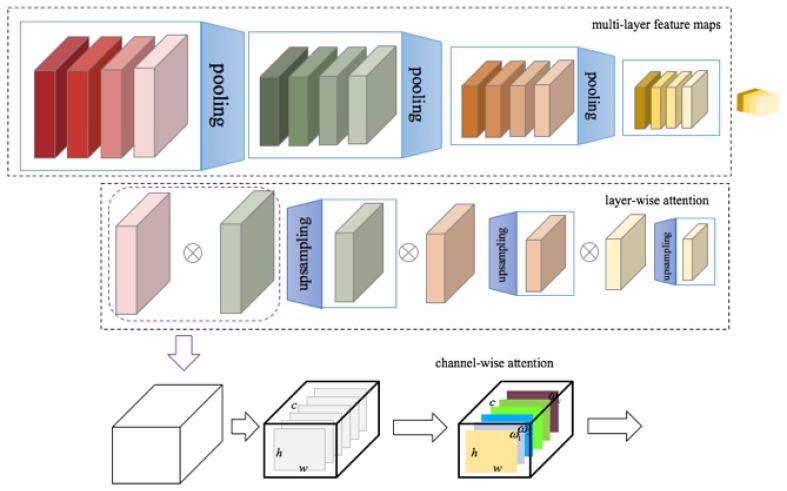
Attention-based network structure. Four layers at different levels have been selected from ResNet-101 for feature fusion; these layers are denoted by $C_{2-5}$ throughout the remainder of this paper.

A model diagram of our proposed feature fusion method based on a multilevel attention mechanism is shown in Figure 8. This model is inspired by a problem decomposition strategy that is usually used for solving complex tasks [56,57,58]. The first layer is a basic feature extraction network, the second layer is layer-wise feature fusion based on the attention mechanism, and the third layer is channel-wise feature fusion based on the attention mechanism.

#### 2.3.3. Feature Layer Selection

The receptive field (RF) refers to the specific features that are being observed in the input space. The value of the RF represents the range of the observable area in the original image. The larger this value is, the more global features the RF may contain. The smaller the value is, the more local features it may contain. ResNet-101 is used as our basic feature extraction network. Because different ResNet-101 layers have different RFs, it is necessary to select appropriate feature layers to obtain appropriate RFs. To illustrate the significance of the RF for detection, consider a traditional five-level CNN as an example: the conv1 RF is too small, capturing little semantic information about potential targets, and using this feature map would increase the number of calculations, whereas the RFs of the conv2 to conv5 features can better cover targets of various sizes, making these feature layers more suitable to use for the task at hand.

By analysing the experimental dataset and data collected from hospitals, we found that the diameters of MAs are mainly distributed between 10 and 40 pixels. The distribution details are given in Table 1. These results were confirmed by an ophthalmologist group from the Eye College of Chengdu University of TCM and were discussed with its members. To enhance the features corresponding to the majority MA size group, i.e., between 10 and 40 pixels, the shallow layers conv2 and conv3 are critical for MA detection. Thus, feature fusion and target detection were performed using the features from layers conv2 to conv5. In the experimental section, the significance of the conv2 and conv3 layers for MA detection is analysed.

#### 2.3.4. Layer-Wise Feature Fusion

As in many other medical data analysis applications [59,60,61], the successful extraction of critical features can effectively improve the performance of the model. To make more effective use of information that is useful for small target detection from different feature layers, an attention mechanism model is introduced in this paper for layer-wise feature fusion. First, the top-level feature map is upsampled to allow it to be merged with the feature map from the previous layer. Specifically, in the process of feature layer fusion, each feature layer is given a weight coefficient, which is initialized to 1; then, the weighted sum of the two feature layers is calculated. Thus, the fused feature map $F_{i}^{w*h*c}$ is calculated as follows:

(1) $$F_{i}^{w*h*c}=\alpha_{1}f_{i}^{w*h*c}+\alpha_{2}f_{i+1}^{\prime w*h*c},$$

where *i* denotes the feature level, meaning that $f_{i}^{w*h*c}$ and $f_{i+1}^{\prime w*h*c}$ denote the feature map from the current layer and the feature map obtained by upsampling the next higher layer, respectively. Feature layer fusion based on the attention mechanism allows the network to determine which features are more important during the detection process and thus to pay more attention to them.

#### 2.3.5. Channel-Wise Feature Fusion

To enhance the useful information for small target detection that is contained among the different channels, this paper also introduces an attention mechanism for channel-wise feature fusion. Global average pooling is applied to $F_{i}^{w*h*c}$ to generate the channel-wise statistic $Z_{i}$:

(2) $$Z_{i}=\mathrm{avepool}\left(F_{i}^{w*h*c}\right).$$

The pooling result $Z_{i}$ is passed through two fully connected layers. Then, the softmax operation is performed. The result is multiplied by $F_{i}^{w*h*c}$ to obtain the merged feature map as follows:

(3) $$R_{i}^{w*k+c}=\mathrm{softmax}\left(\delta \left(W_{1}\delta \left(W_{1}Z_{i}\right)\right)\right)⊙F_{i}^{w*h*c_{*}},$$

where δ denotes the ReLU activation function [62], $W_{1}\in R^{c\cdot \frac{c}{8}}$, and $W_{2}\in R^{\frac{c}{8}\cdot c}$.

#### 2.3.6. Loss Function

To optimize the small object detection model, the loss function is defined to consist of two terms: a detection loss and an attention loss:

(4) $$\mathrm{Loss}=L_{\mathrm{detection}}+\lambda L_{\mathrm{attention}}.$$

$L_{\mathrm{detection}}$ is the loss function for the predicted bounding box position and the object score in object detection, and $L_{\mathrm{attention}}$ is the loss function for feature fusion based on the attention mechanism. The hyperparameter λ controls how much weight is assigned to the attention mechanism.

The object detection loss function further consists of two main terms: one for correcting the predicted bounding box location and one for correcting the object score in the predicted box. The specific formula is as follows:

(5) $$L_{\mathrm{detection}}=\frac{1}{N^{L}}\sum_{k=1}^{N^{L}}L_{loc}\left(\theta |B_{k},T_{k},R_{k}\right)+\frac{1}{N^{O}}\sum_{k=1}^{N^{o}}L_{obj}\left(\theta |B_{k},T_{k},R_{k}\right),$$

where θ refers to a learnable network parameter and $\left\{B_{k},T_{k},R_{k}\right\}$ is a training triple used to learn the target location and target score. To minimize the objective function, random gradient descent optimization and training triples collected via image-centred sampling are used.

In the multilayer feature fusion attention loss function, by comparing the predicted score map with the ground-truth object map, we can determine which pixels are more likely to include objects and thus require more attention; then, the weights can be further adjusted:

(6) $$L_{\mathrm{attention}}={∥X-D^{GT}∥}^{2},$$

where *X* denotes the predicted score map, and $D^{GT}$ is the ground-truth object map. The predicted score map is filled with the corresponding confidence scores of the predicted bounding boxes on the graph, and the remaining areas are then filled in with a fixed value of 0.05.

#### 2.3.7. Secondary Screening Based on Spatial Confidence

Inspired by [63,64], the environmental context of the targets can assist in the detection of MAs. An MA is a roughly spherical object with normal vascular eminence, or a small blood dot that is formed by the expansion and leakage of a blood vessel; thus, MAs typically appear near blood vessels. Based on experimental statistics, the corresponding distance distribution is shown in Table 2. The distances between MAs and blood vessels can serve as a reference for discriminating MAs.

The first step is to calculate the distance between each predicted bounding box and the nearest blood vessel in the preliminary detection results. The distance calculation algorithm is presented in Algorithm 1.

**Algorithm 1:** Distance Calculation Algorithm.**Input:** Vascular segmentation map *I*; Candidate object set $S=\left\{\left(x_{1}^{i},y_{1}^{i}\right)\left(x_{2}^{i},y_{2}^{i}\right)|S_{i}\right\}$;

**Output:** Candidate object and vascular distance set *D*;
1:Initialize distance set D=Φ, initial distance $d_{i}=0$;2:Judge whether blood vessels appear in the region $\left(x_{1}^{i},y_{1}^{i}\right)\left(x_{2}^{i},y_{2}^{i}\right)$;3:If yes, add the distance $d_{i}$ to set *D* and reset $d_{i}$ to 0. Select the next candidate box and return to step 2. If no next candidate box exists, terminate the algorithm;4:Otherwise, set $x_{1}^{i}=x_{1}^{i}-5$, $y_{1}^{i}=y_{1}^{i}-5$, $x_{2}^{i}=x_{2}^{i}+5$, and $y_{2}^{i}=y_{2}^{i}+5$ and determine whether the coordinates overflow. If $x_{1}^{i}<0$ or $y_{1}^{i}<0$, then set $x_{1}^{i}=0$ or $y_{1}^{i}=0$. If $|x_{2}^{i}>W$ or $y_{2}^{i}>H$, then set $x_{2}^{i}=W$ or $y_{2}^{i}=H$;5:$d_{i}=d_{i}+5$, return to step 2;

Once the distance between each predicted bounding box and the corresponding blood vessel has been obtained, a majority voting mechanism is introduced in this paper for screening the preliminary detection results. The confidence score of each predicted bounding box is calculated according to the following formula, which represents the probability that the corresponding object is an MA. $C_{p}$ denotes the critical distance, and $C_{i}$ denotes the spatial confidence score for the current box:

(7) $$C_{i}=\frac{C_{p}-{\mathrm{distance}}_{i}}{C_{p}}.$$

Finally, according to the category probability score and the confidence score of each predicted box, the voting mechanism is used to screen out some false positive objects that fall either on or at too far a distance from blood vessels to improve the detection accuracy. The majority voting function is formulated as follows:

(8) $$S\left(t\right)=\sum \left(P_{\mathrm{class}}*\alpha +C_{t}*\beta \right).$$

This function calculates a weighted average of the category probability score from the preliminary detection of the candidate MA, denoted by $P_{\;\mathrm{class}\;}$, and the confidence score of the candidate MA, denoted by $C_{t}$, using appropriate weights α and β (we set α=0.9 and β=0.1). The structures of the two models used in this paper are obviously different; together, they comprehensively detect and distinguish MAs from different perspectives and improve the accuracy of the final detection results.

## 3. Results and Discussion

### 3.1. Configuration

The experimental design are as follows. In the experiments, we first compared existing object detection algorithms with the attention-based feature fusion method proposed in this paper and then compared the fusion results of schemes involving different layers based on the attention mechanism. Then, the confidence of the preliminary detection results based on the proposed feature fusion method was judged, and the validity of the confidence degree was assessed based on the accuracy of the detection results. Finally, we compared our method with other MA detection methods.

The parameters related to the experiments are listed in Table 3. The learning rate represents the speed of parameter updating; the momentum is the weight of the previous gradient update during the gradient update process and protects the model from both the disappearing and exploding gradient problems. Gamma is the weight parameter for the learning rate strategy; Weight_decay is the weight of the attenuation rate, which is used to prevent overfitting; Batch_size is the number of images read each time; Num_seed_boxex is the initial number of seed frames generated; and Num_output_boxes is the final number of seed frames.

To comprehensively evaluate the effectiveness of the proposed method, the following metrics were adopted: precision, recall (sensitivity), average precision, and F-measure. Before the above evaluation indicators can be defined, several related concepts must be introduced: the number of true positives (TP) is the number of positive samples that are correctly identified as positive samples, the number of true negatives (TN) is the number of negative samples that are correctly identified as negative samples, the number of false positives (FP) is the number of negative samples misidentified as positive samples, and the number of false negatives (FN) is the number of positive samples misidentified as negative samples. The Intersection-over-Union (IoU) reflects the degree of coincidence between the detection result of the model and the original ground-truth frame, and it is calculated as follows:

(9) $$IoU=\frac{DR\cap GT}{DR\cup GT}.$$

Among the specific evaluation indicators, the precision refers to the proportion of positive samples among the detected results and is calculated as follows:

(10) $$\;\mathrm{Precision}\;=\frac{TP}{TP+FP}.$$

The recall rate (sensitivity) is the proportion of positive samples detected among all positive samples and is calculated as follows:

(11) $$\;\mathrm{Recall}\;=\frac{TP}{TP+FN}=\mathrm{Sensitivity}.$$

The average precision (AP) is the area under the precision–recall curve. In this paper, the 11-point calculation method used in the Pascal VOC2007 challenge is applied to calculate the AP. The precision is measured at 11 different points according to the recall interval, and the calculation formula is as follows:

(12) $$AP_{\mathrm{Ipoint}}=\frac{1}{11}\sum x\;\left(x\in \mathrm{MaxPrecision}\right).$$

The F-measure is a comprehensive evaluation index that comprehensively considers the discrepancy between precision and recall; specifically, it is the weighted harmonic average of the precision and recall. This paper mainly uses the F1 score, which is calculated as follows:

(13) $$F1=\frac{2}{1/\;\mathrm{precision}\;+1/\mathrm{recall}}.$$

In this paper, three object detection methods, namely, SSD, Faster R-CNN and AttractioNet, are used as the base algorithms for comparison. SSD is a regression-based target detection algorithm, while Faster R-CNN and AttractioNet are based on region-nomination target detection algorithms. By comparing SSD with Faster R-CNN and AttractioNet, the adaptability of these two types of detection algorithms to fundus images can be verified. By comparing Faster R-CNN with AttractioNet, it is possible to verify the adaptability of different region nomination algorithms (an RPN and an Attend & Refine Network, ARN) to fundus images.

The specific experimental comparison design is as follows. First, SSD, Faster R-CNN and AttractioNet were compared with our feature fusion method (Ours-1) based on the attention mechanism proposed in this paper. Then, the preliminary detection results obtained with the proposed attention-based feature fusion method were compared with the further results obtained with the spatial confidence discrimination method (Ours-2) proposed in this paper.

### 3.2. Experimental Results

All experiments were conducted using the IDRiD_VOC dataset described in Section 2.1. The performances of the different detection algorithms are as follows.

For the experiment with the proposed feature fusion method based on the multilayer attention mechanism, Table 4 compares the detection results of the existing target detection algorithms with those of the proposed attention-based feature fusion method using the AP metric. Table 5 compares the results of fusion schemes using different layers $C_{i}\left(i=2,3,4,5\right)$ for attention-based feature fusion using the precision, recall rate and F1 score as the evaluation indicators.

For the experiment conducted to test the confidence discrimination method based on spatial confidence proposed in this paper, Table 6 shows the detection results obtained by applying the confidence degree based on the preliminary test results obtained with Ours-1, using the detection precision and sensitivity as the evaluation indices.

To illustrate the performance of our methods, the results of various object detection methods are compared.

Table 4 compares the detection results of the existing object detection algorithms with those of the proposed feature fusion method based on the attention mechanism. At different IoU thresholds, our algorithm outperforms its competitors based on the corresponding AP metrics. When the IoU threshold is 0.5, the AP value of our algorithm reaches 0.757.

In Table 4, the Ours-1 method is applied to fuse downsampled features from the C5 layer and previous layers based on the attention mechanism. Considering that shallow layers capture rich feature information relevant to small targets, the appropriate layers $C_{2}$, $C_{3}$, $C_{4}$, and $C_{5}$ are selected for feature fusion. In terms of the AP metric, Ours-1 achieves better detection performance than the other detection algorithms do. SSD also uses multilayer features for prediction. However, most of the feature information that is useful for small targets is contained in the shallow layers. Ours-1 uses the shallow layers $C_{2}$, $C_{3}$, $C_{4}$, and $C_{5}$, and thus can effectively extract features that are relevant to small targets, while SSD uses the deeper layers $C_{8-11}$, which may result in the absence of small target features. Although SSD also uses layer $C_{4}$, a single layer is a weak basis on which to extract small target features; by contrast, Faster R-CNN and AttractioNet use region nomination networks and thus achieve better accuracy than SSD for small target detection. Nevertheless, Ours-1 uses layers $C_{2}$, $C_{3}$, $C_{4}$ and $C_{5}$ to extract abundant features relevant to small targets, while Faster R-CNN and AttractioNet use only the top-level features of layer $C_{5}$ for prediction and thus ignore crucial shallow-layer features for small object detection. Thus, Ours-1 is superior to SSD, Faster R-CNN, and AttractioNet.

Table 5 compares the results of using different layers $C_{i}$(i=2,3,4,5) for feature fusion based on the attention mechanism. As seen from this comparison, the inclusion of shallow features effectively improves the MA detection performance. For the base fusion scheme (using only $C_{4}$ and $C_{5}$) with attention, the F1 score is 0.582, whereas the F1 score increases to 0.840 when $C_{2}$ and $C_{3}$ are added.

Table 5 compares the results of different fusion schemes using the proposed attention-based feature fusion method. The results without the attention mechanism are also included in Table 5 for the fusion of the same feature layers. The experimental results show that the attention mechanism effectively improves the detection precision and recall rate for a given fusion scheme. For example, when only $C_{4}$ and $C_{5}$ are considered for feature fusion, the F1 score increases from 0.549 to 0.582 when the attention mechanism is adopted. For fusion schemes at different levels, when shallower feature layers are used, a higher detection recall rate is achieved. This finding shows that smaller objects are better detected when features from shallow layers are included because these features are more conducive to the detection of small targets. However, the semantic information of shallow feature layers is not sufficiently rich, leading to a decrease in precision. For instance, the detection precision achieved when features from $C_{3}$, $C_{4}$, and $C_{5}$ is higher than that achieved when using features from $C_{2}$, $C_{3}$, $C_{4}$, and $C_{5}$; however, the comprehensive F1 score increases in the latter case, indicating that the overall detection performance is improved.

Table 6 compares the preliminary detection results obtained with Ours-1 and the further results obtained when confidence discrimination is applied. When the voting function threshold is set to 0.8, the precision of Ours-2 reaches 0.895.

In Table 6, the preliminary test results of Ours-1 are compared with those of the confidence discriminant method. Under different probability thresholds, the influence factors are the accuracy and sensitivity of detection. The experimental results show that higher sensitivity can be obtained with lower probability threshold, but its accuracy decreases. As a discriminant factor, spatial confidence can reduce the misjudgment of pseudoaneurysms on or far from blood vessels. The strategy improves the accuracy of the model (by 2–4 percentage points) while maintaining the sensitivity of the model.

**Figure 9:**
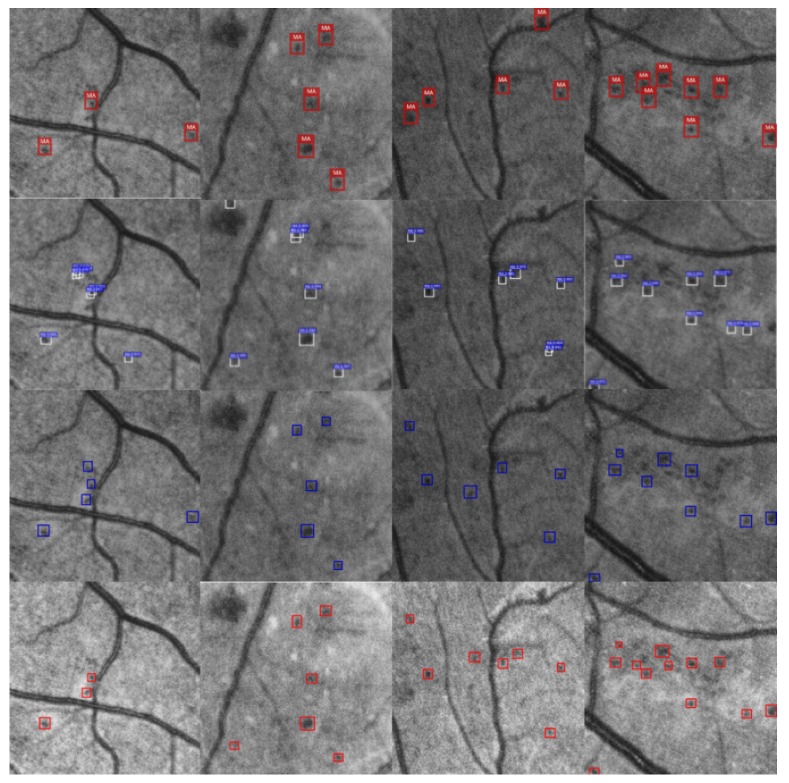
Detection results with an IoU threshold of 0.5. The first row corresponds to SSD, the second row shows the detection results of Faster R-CNN, the third row shows the detection results of AttractioNet, and the last row shows the results of our method, which performs the best on this dataset.

**Figure 10:**
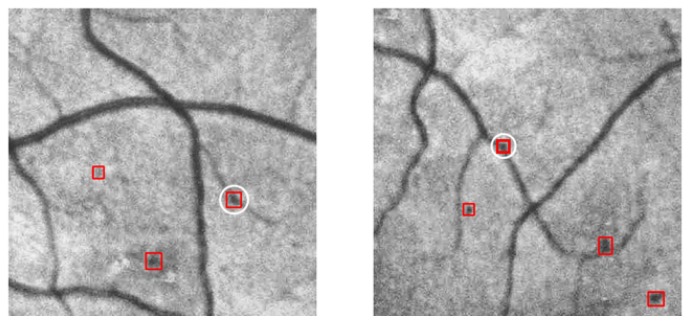
Screening out false detections using spatial confidence. Instances showing an excessive distance between the target and the nearest blood vessel are screened out. The two examples shown in this figure represent another typical source of false detections with our method. These areas are treated as target objects because some pixel points on a blood vessel are darker than their surrounding pixels. Such mistakes primarily occur due to the imaging environment and can easily lead to a low $P_{class}$ but a high $C_{t}$; therefore, we set α=0.9 and β=0.9 to avoid such false detections.

Selected detection results from the experiment are shown in Figure 9 and Figure 10. Figure 9 compares the detection results of Ours-1 with those of the other models based on contrast detection. The first row shows the detection results of SSD, the second row shows the detection results of Faster R-CNN, the third row shows the detection results of AttractioNet, and the fourth row shows the detection results of Ours-1. Figure 10 shows examples of false detection based on confidence degree discrimination in which the falsely detected objects fall on a blood vessel. The white circles indicate such false detections. The distance between such an object and the nearest blood vessel is obtained by calculating the distance relationship, and the corresponding confidence score is 0. Through majority voting, these false objects can be effectively screened out, thereby improving the detection accuracy.

Based on the small object detection experiments reported in this paper, Table 7 compares the method proposed in this paper with other methods for MA detection, using the detection sensitivity as the evaluation index. As shown, our method achieves the highest sensitivity value of 0.868.

As shown in Table 7, MA detection algorithms based on different protocols were selected for an experimental comparison. Here, because the sensitivity was chosen as the indicator, the experimental results are compared with those from [30], in which a single network is used to detect multiple lesions such as exudate, hemorrhage, and MA regions, and [31], in which blood vessel removal and re-extraction are first performed. In addition, the methods of [32,33] and others detect MAs through pixel-by-pixel classification. This comparison shows that the proposed approach can more successfully detect MAs in fundus images.

## 4. Conclusions

Automated MA detection in fundus images can effectively ease the demand on medical resources. A novel deep learning framework for MA detection is proposed in this paper. This framework is based on an attention mechanism and on the relative spatial distribution of blood vessels and MAs. Shallow-layer network features related to small target detection receive special focus due to our attention module, leading to better performance compared with competing methods. Afterwards, a spatial voting method is applied to further improve the detection results. The experiments conducted in this study confirm the performance of the proposed method based on the IDRiD_VOC dataset, which was collected from the open IDRiD dataset. The proposed method achieves better detection performance than the other tested methods and constitutes a valid approach for MA detection.

The fundus images considered in this study were captured by only one type of fundus camera. Different cameras may introduce new features or produce images with different layouts. Therefore, future studies should include more images captured by different types of fundus cameras to improve the generalization ability of the proposed model. 

## Figures and Tables

**Table 1:** Microaneurysm size distribution.

**Diameter (Pixels)**	<5	5–10	10–20	20–30	30–40	40–50	>50
**Percentage**	0.02%	1.58%	47.40%	39.70%	9.80%	1.2%	0.30%

The diameter range of 5–40 pixels contains more than 90% of the examples and corresponds to shallow features.

**Table 2:** Microaneurysm and blood vessel distance distribution.

**Distance (Pixels)**	0–10	10–20	20–30	30–40	40–50	50–100	100∼200
**Percentage**	80.70%	7.60%	4.20%	1.30%	2.20%	2.20%	1.80%

The data in this table reflect the mathematical spatial characteristics of MAs to a certain extent, and the distances between MAs and blood vessels can be used as a reference to discriminate MAs.

**Table 3:** Overview of the related parameters.

Parameter	Value
Learning rate	0.001
Momentum	0.9
Gamma	0.1
Weight_decay	0.0001
Batch_size	64
Num_seed_boxex	10000
Num_output_boxes	2000

**Table 4:** Detection results of different methods.

Method	Proposals	Features	Fusion	Attention	${\mathrm{AP}}^{50}$	${\mathrm{AP}}^{60}$	${\mathrm{AP}}^{70}$
SSD	–	$C_{4}$,$F_{C_{7}}$,$C_{8-11}$	✓	–	0.582	0.473	0.231
Faster R-CNN	300	$C_{5}$	–	–	0.684	0.515	0.269
AttractioNet	2000	$C_{5}$	–	–	0.721	0.517	0.264
Ours-1	2000	$C_{2}$,$C_{3}$,$C_{4}$,$C_{5}$	✓	✓	0.757	0.523	0.321

The superscript of AP represents the IoU threshold corresponding to the indicator. For example, $AP^{50}$ means that the IoU threshold is 0.5.

**Table 5:** Detection results when fusing different layers.

Ours-1	Attention	Precision	Recall	F1
$C_{4}$+$C_{5}$	–	0.793	0.420	0.549
	✓	0.833	0.447	0.582
$C_{3}$+$C_{4}$+$C_{5}$	–	0.823	0.591	0.688
	✓	0.876	0.639	0.739
$C_{2}$+$C_{3}$+$C_{4}$+$C_{5}$	–	0.801	0.763	0.782
	✓	0.872	0.810	0.840

As the low-level feature layers $C_{2}$ and $C_{3}$ are integrated, the model performance gradually improves.

**Table 6:** Detection results with confidence discrimination.

	0.4		0.6		0.8	
	Precision	Sensitivity	Precision	Sensitivity	Precision	Sensitivity
Ours-1	0.831	0.868	0.851	0.849	0.870	0.823
Ours-2	0.874	–	0.885	–	0.895	–

The numbers in the first row correspond to the voting function threshold. An improvement in precision results in a decline in sensitivity.

**Table 7:** Results of MA detection.

Method	Dataset	Sensitivity
Tan [30]	CLEOPATRA	0.461
Dai [31]	ROC [65]	0.691
Khojasteh [32]	DIARETDB1 [66]	0.85
Adal [33]	DIARETDB1 [66]	0.646
Ours	IDRiD_VOC	0.868
